# The brain, self and society: a social-neuroscience model of predictive processing

**DOI:** 10.1080/17470919.2018.1471003

**Published:** 2018-05-10

**Authors:** Michael P Kelly, Natasha M. Kriznik, Ann Louise Kinmonth, Paul C. Fletcher

**Affiliations:** a Department of Public Health and Primary Care, Institute of Public Health, University of Cambridge, Cambridge, UK; b THIS Institute (The Healthcare Improvement Studies Institute), University of Cambridge, Cambridge Biomedical Campus, Cambridge, UK; c Department of Public Health and Primary Care Institute of Public Health, Fellow St Johns College University of Cambridge, Cambridge, UK; d Department of Psychiatry, University of Cambridge, Cambridge, UK

**Keywords:** Predictive processing, typifications, ideal types, social practice, social behavior, brain models, intersubjectivity

## Abstract

This paper presents a hypothesis about how social interactions shape and influence predictive processing in the brain. The paper integrates concepts from neuroscience and sociology where a gulf presently exists between the ways that each describe the same phenomenon – how the social world is engaged with by thinking humans. We combine the concepts of predictive processing models (also called predictive coding models in the neuroscience literature) with ideal types, typifications and social practice – concepts from the sociological literature. This generates a unified hypothetical framework integrating the social world and hypothesised brain processes. The hypothesis combines aspects of neuroscience and psychology with social theory to show how social behaviors may be “mapped” onto brain processes. It outlines a conceptual framework that connects the two disciplines and that may enable creative dialogue and potential future research.

## Introduction

We argue that two social processes play an important part in prediction-based inference in the human brain. First, physical interaction between individuals and between individuals and their physical and biological environment has to happen. Second, humans have to exercise their capacity to share their subjectivity with others – intersubjectivity (Kelly & Kelly, 2018). Predictive processing is the computational process by which a brain can model its external world and its internal feeling state; it makes inferences about the causes of the inputs it receives. Those predictive processes include ideal types and typifications arising in the social practices of everyday life. We show how the prediction-based generative modelling, that has been described by neuroscientists (Clark, 2016; Frith, 2007; Pezzulo, Rigoli, & Friston, 2015; Seth, 2015) is linked through the concept of the self to ideal types, typifications and social practices, concepts which have been defined by sociologists (Giddens, 1979, 1982, 1984; Mead, 1934; Schutz, 1964, 1970; Shove, Pantzar, & Watson, 2012; Weber, 1949).

## The brain as a model of the world

We begin with an account of the processes occurring within the individual brain and then go on to consider the interactions between the brain and the external social environment. Our initial proposition is that the brain is a model of the external world (cf Conant & Ashby, 1970). The concept of the brain as a model of the world (Frith, 2007), and the associated idea of predictive processing are based on the principle that the brain develops its model of the world by engaging in generative modelling – reasoning backwards from sense data to infer what external events and stimuli are most likely to have caused these sense data. It does this on the basis of *a priori* models (Clark, 2016; Frith, 2007; Pezzulo et al., 2015; Seth, 2015). Ideal types and typifications formed in social interaction are intrinsic to those *a priori* models because they help encode some sense data. We focus therefore below on the social interaction between the person and their external environment, and the resulting generative modelling

The world is a changeable place. Both automatic and planned actions (Strack & Deutsch, 2004) take advantage of and mitigate the impact of potentially harmful changes in the external environment. This allows for the exertion of some degree of control over the factors that could threaten organism stability. In anticipating these changes and reacting to them, the brain works as a regulatory system which acts by making a model of its world (Conant & Ashby, 1970). A key, and long-standing question in neuroscience is how can it do this when the signals it receives from that world are noisy, inconsistent and ambiguous? One answer is that it deals with the noise and ambiguity through predictive processing. This means that it uses prior knowledge to interpret these signals in order to infer the most likely causes of the inputs. Such a system requires experience and sensory input as well as, crucially, a capacity to integrate the two in pursuit of optimal modelling. Incoming evidence and other signals that do not accord with current expectations will produce a prediction error. The error in turn can form the basis for updating the model (Frith, 2007: 134–6). Alternatively the prediction error may be ignored especially if it is weak or unreliable. If updating occurs, the next set of predictions are based on that updated model (Clark, 2016:13–52).

A central problem for the inferring brain is that it does not have direct contact with the causes of sensory inputs in its world. It has only all the various ambiguous signals, both internal to the body (e.g. blood pressure, heart rate) and external to the body (e.g. vision, sound, touch). Its solution is to make generative inferences (i.e. to infer the causes of those inputs) and by predictive processing to use these as the basis for predicting future inputs. The central idea of predictive processing is that in order to make sense of ambiguous noisy inputs, the brain makes use of predictions based on prior experience. But predictions can be inaccurate, producing prediction errors. A policy of minimising such errors should therefore ensure optimal future inferences in a changing world. However, given that some degree of inaccuracy of prediction error is inevitable, the dilemma arises over when to update in response to errors and when to ignore them. We develop this idea below.

Our conscious experience of the world, therefore, is largely a prediction of what is about to happen next or, more accurately, what the next inputs are likely to be (Clark, 2016: 168–71; cf Schutz, 1970:67). This not only works for interpreting incoming data but it also drives actions and movements. So future motor intentions are also subject to future predictions. Thus, an action becomes something that occurs to fulfil the prediction that happens as a consequence of having a motor intention (Frith, 2007:106). That is, we can view actions as occurring in response to the mismatch between current state and motor prediction. As such an action can be conceived as occurring to minimise a prediction error. This perspective has been extended to a consideration of emotion and feeling as arising from similarly prediction-based inference about bodily states from an array of internal signals (Seth & Critchley, 2013). The predictive framework offers an integrated perspective on action, perception, emotion and cognition (Clark, 2016: 4–7; 68–9). Consideration of all these ideas in detail is beyond the scope of this paper and we focus primarily on their implications for social interactions and inferences.

The critical point in respect of social interaction is that humans are able to do much more than predict their immediate movements and actions. They can predict and imagine their own and others’ future and past actions and intentions too. The machinery for prediction that subserves perception and controls actions of the individual extends to anticipating and interpreting other people’s actions (Clark, 2016: 139–151; cf Mead, 1934:147–159). So at the same time as the human can predict their own simple movements, like waving a hand or bending a finger, they can simultaneously engage with a cultural universe with a past, a present and an anticipated future. They can also anticipate the actions of other people and things, not only in their present purview, (the people and things that they can see in their immediate fields of vision and experience) but they can anticipate and reason about social and cultural worlds past, present and future (Zerubavel, 1997:7). They have a highly developed awareness of themselves in relation to those worlds.

Active inference is the minimisation of prediction errors through performing actions in the service of intentions (Seth, 2015: 18–19). The essence of active inference is that movements/actions are generated by prediction errors (a mismatch between intention and experience). They are performed in the service of intentions that aim at minimising those prediction errors. Consequently the action does not seek to disconfirm but rather attempts to confirm the current prediction (the intention). As well as physical movements, these inferences drive physical and symbolic (i.e. imaginative) interaction with others (cf Blumer, 1962). Active inference is the combined mechanism by which perceptual and motor systems work together to reduce prediction error using the twin strategies of altering predictions to fit the world and altering the world to fit predictions (Clark, 2016: 122). In other words, individuals act on the world to make their model reality; they are not just passive subjects being acted on or determined by the world

Good inferences will make better predictions and so prediction error becomes a valuable marker for the validity of the current model as well as an important drive to model updating. Not all brains accomplish this optimally and as we argue below, the capacity to optimise updating is differentially distributed across the population. Through the iterative process of inferring, predicting, error-monitoring and updating, the brain’s model is developed and each individual will create models differently. Given that different individuals create models that are distinct from one another, the individual brain faces two immediate tasks; the first is to orient its own behavior in line with its developing model and the second is to ascertain the degree to which its models of the world align with those of others in its immediate and wider environments. So at once, there is both a neuroscientific computational intra-individual problem and a problem that has to be solved inter-subjectively in order for social interaction to proceed. The latter is accomplished in part through the processes of socialisation from infancy through childhood and continuing into adult life, which in turn interact with the neuroscientific processes. It is also in part facilitated through the use of ideal types and typifications which we describe below.

For the computational neuroscientific problem, at its simplest, the brain has to focus on minimising prediction error for the best possible model to emerge. But prediction errors are inevitable since the senses are limited, models are always simplifications and since the world is noisy, uncertain and changing. Humans need a means to deal with this. The question of when to update in response to prediction error and when to ignore this error and retain previous inferences is therefore critical. If the model does not align with new information, the organism finds itself maladapted to its environment (Pezzulo et al., 2015), but if it updates too readily it will over-fit the data and miss statistical regularities that are important and real, albeit probabilistic. The problem is one that attends any modelling enterprise: the model must be a pragmatic simplification and so is inevitably partly wrong (Clark, 2016: 69–71) but it can be rectified. An unexpected response can signal that the prediction is wrong but does not necessarily signal that it would be optimal to change or fix it (Fletcher & Frith, 2009).

In summary, the brain may be considered a predictive inference device, integrating both external and internal signals with prior knowledge in order to infer the likely causes of those signals and thereby to model, and ultimately regulate, the world. Prior experience helps to stabilise error response. *Prima facie*, this is a reductionist perspective, but actually this model puts a powerful emphasis on the environment and the social, as well as the individual, as facilitators of brain processes (Franks, 2010:, 2010: 59). In recognising that the brain functions as a model of the world, we are acknowledging that brain processes may be understood in the context of the world that the brain inhabits; its body and the world beyond. The predictive processing model therefore demands conceptualisations of the brain at levels that go beyond the neurobiological and cognitive to the social (cf Franks, 2010: 39). We next elucidate the nature of the interaction between the self and the external environment.

## The self

For Seth the self is the critical locus of the interaction between the brain and the social world (Seth, 2015). We concur. Our conception follows Kant who described the self in *the Critique of Pure Reason* (Kant, 1781/1787/2007) (pre-figuring predictive processing) by arguing that humans make judgements in order to make sense of the world. They do this on the basis both of empirical sense data and *a priori* categories derived from previous experience (cf Zerubavel, 1997: 2–5). Kant also identified the self as the place where such judgements are made (cf Frith, 2007:138). Historically these Kantian ideas were central to the conceptions of self, which were developed in the philosophy and social psychology of William James (1892) and George Herbert Mead (1934), in the phenomenological writings of Alfred Schutz (Schutz, 1964:20) and in sociology in symbolic interactionism (Blumer, 1962; Stone & Farberman, 1981). We draw on these insights to develop our account.

We conceptualise the self as a thinking subject, who knows that they are thinking, who can reflect, ruminate and act on the external world and is aware of their capacities for reflexivity and agency. That external world is an empirical totality of potential sensory inputs (Kelly, 2015). The self is the conscious awareness of the process of actively interpreting external and internal information and stimuli. In doing so it conceives of itself as a subject separate from the physical external world and from other people and as existing through time and being situated in place. It is able to think of its self as a self-directing agent – an “I” – with an autobiography expressed in the language forms “I do”, “I will do”, “I can do”, “I have done” and so on. The self and the “I” enable predictive processing to move from being about simple motor responses and intention to engaging with the broader social and cultural universe. That self is the centre of its own experiences of that cultural universe.

The brain predictively processes external inputs and internal subjective experience consisting of the experience of the body (Seth, 2015:9–10) and of consciousness, in order to maintain homeostasis. The experience of the body, of one’s consciousness and of being in the wider world are the platforms on which the social self develops. The self is social because it arises and develops in social interaction (Mead, 1934). It is only possible to have a self if one has experience of interaction with others – it is quintessentially relational. The self has a dual aspect. First, it is substantial and experienced as something unitary across place and time in the sense that “I am the same person I was yesterday, last week, last year or when I was a child”. Second, it is also situational and experienced as something in the here and now intrinsically linked to current and immediate actions and roles, say managing the acute pain I am currently suffering or concentrating on the book I am reading at the moment. These second multiple situational aspects of self may be experienced as dissociated or partly disconnected from my experience of other situational selves – the self I was before I was in pain or when I was eating my breakfast earlier this morning. They may also be experienced as if they disjoined from the substantial idea of self (Kelly, 1992; Kelly & Dickinson, 1997; Turner, 1968). Both aspects of self – the substantial and situated – arise in relational interactive processes. Both are experienced as something unique to the isolated individual and linked to the individual’s body. Given that the brain’s access to its own body is, just as with external stimuli, noisy and ambiguous, the way that the body is experienced and the arising sense of self depends on the brain’s inference of what the causes of the internal and external sensory signals it is experiencing are most likely to be (Seth, 2015:11; Frith, 2007:126–7).

## Social life

Echoing Mead and Schutz and linking to more recent social theory (Giddens, 1979, 1982, 1984) we suggest that the interfaces between the brain, the self and society and the intra-individual nature of the neuroscientific processes described above and the development of self may be understood as follows. Human social life – society – has two characteristics. First, it changes continuously, sometimes very gradually, sometimes seismically. Second, it also has a repetitive and recursive quality (Giddens, 1984: xxiii, 17–19). Most people most of the time engage in more or less the same activities and practices on a day-to-day basis. Therefore much human activity can be, in Schutz’s words, “taken for granted” (Schutz, 1970: 79–80; cf Clark, 2016: 54–8) and therefore successfully accomplished without the human having to do much other than go with the flow of interaction and engage in habitual activities (Strack & Deutsch, 2004; : Kahneman, 2011). Extant brain models work well in these circumstances because this repetitive and recursive social reality requires only minimal model adjustments. Predictions work accurately enough most of the time; prediction error is marginal and therefore the models need only slight updating. Not much is required internally by way of remodelling the external world and the prior predictive models do not need to be fundamentally changed.

Human life as well as being recursive and repetitive, also throws up considerable novel challenges. Challenges arise because the signals from the external environment are ambiguous, unclear or discordant from the perspective of the brain’s existing model of the world. As noted above, no model can be unerringly accurate in its predictions. Therefore, when the signals do not align with the models especially when they are highly discordant, the brain has to engage in significant reflective remodelling. However, and this is fundamentally important, this does not cause most people much distress or difficulty most of the time; embarrassing as it is, making *faux pas* during social interaction and correcting behavior for the future, is commonplace, normal and routine. Moreover most adult humans most of the time are highly adept in social interaction at knowing when to overlook or ignore the *faux pas* of others in order to allow interaction to proceed smoothly (cf Garfinkel, 1967; Goffman, 1969). The processes of adjustment happen quickly. Even where the signals require active cognitive engagement most people, at least by the time they reach their teens, are very skilled at adjusting their predictive models to such an extent they may not even consciously acknowledge that it has happened. They also can comfortably hold differing models as workable at the same time (Festinger, 1957).

New and unfamiliar circumstances or environments, major life changes, events and experiences and the emotional and hormonal reactions to the unfamiliar or to the changes themselves also present challenges to the brain. In adjusting to major challenges two things happen germane to our argument. First, the excretion of stress hormones such as cortisol – there is a physical reaction with physiological consequences which are signals to the brain. Second, humans exhibit enormous capacity to adjust, adapt and cope with such challenges (Lazarus & Folkman, 1984) and part of that process involves the remodelling by the brain.

## Ideal types and typifications

There are two aspects of the way that people navigate and make sense of the web of recursive and familiar and the novel and challenging social practices which they encounter. First, humans draw upon stocks of knowledge – priors- from past social experience or acquired vicariously by listening to and hearing what other people say and describe, and via access to media in all its forms (Di Maggio, 1997: 267). Second, in making sense of their stocks of knowledge and new stimuli, humans use what Schutz called ideal types and typifications to build new models or to redesign old models. In the course of daily life, people access the stocks of knowledge that they have to hand. These serve as a framework of interpretation of past and present experience and anticipation of things to come (Schutz, 1970:74). Some of this knowledge is clear and consistent (albeit provisional and sometimes wrong). Some is vague, obscure and ambiguous (Tversky & Kahneman, 1974: 74). Knowledge is not homogeneous; it tends to be incoherent, partially clear and certainly not free from contradictions (Schutz, 1970: 75). Using ideal types helps solve this problem.

Ideal types are based on idealised notions of what is out there in the external world. Ideal types consist of generalized assumptions about how the world is and how it works. Ideal types are high level organizing priors which are revised and refined based on experience, direct or indirect (Schutz, 1967: 185). Ideal types function as grand schematic priors and allow us broadly to categorise and organise the multiplicity of ambiguous stimuli, which surround us. Typifications are derived from these and are used to interpret that which we see against ideal notions of what we expect the world to be like (cf Weber, 1949). Typifications, which are plastic, arise in intersubjective interaction and provide the basis for signalling internally to the brain (cf Di Maggio, 1997: 267, 276; Rosch, 1978).

So in order to understand the world of immediate and vicarious experience humans begin by using ideal types (Schutz, 1967:184). Ideal types do not necessarily exist in reality; rather they are a convenient inter-subjectively shared way of imagining and then beginning the process of organising complex observations quickly before typifications are engaged to refine understanding progressively. Transforming ideal types into typifications involves regressive inference – reasoning backwards from the sense data. The grand prior or schematic is the generalised ideal type which allows us to work backwards and top down in the typification process to understand with greater precision what we think we are observing (Schutz, 1967:191). Typifications allow for the development of precision to match observation. We inhabit a world of typical objects and practices because of the recursive and repetitive nature of human life. So roles, statuses, situations, institutions are all experienced as typifications (Schutz, 1970: 118–9) and the general principles are contained in ideal types of these typical things. Typifications are an assemblage of the elements to develop the building blocks of brain models. The higher-level ideal types help to direct one to the lower level detailed typifications. Typifications are used to and evolve as the person makes sense of complex/ambiguous inputs. They arise in the course of social interaction and they act as the medium through which signals are encoded. The typifications are aspects of external and internal world, which the brain uses; the typifications generated by the self are a microcosm of the macrocosm – the wider world (Shalin, 1984: 43). Typifications are the way in which we bridge the gap between what we observe and our past knowledge and expectations, they are the basis of the way we make judgements, how we draw together ideas and experience and so they are the basis of predictive processing (cf Schutz, 1970:274–5).

Typifications are not fixed. They are malleable and can be adjusted quickly as the need arises. They can evolve on the basis of new information and provide the means of organising phenomena in the external environment as they appear to be (rather than as they really are) that is good enough most of the time to ensure, for most people most of the time, that interaction with others and the physical environment can proceed reasonably satisfactorily. So typifications are not coherent realist ontologies (Arp, Smith, & Spear, 2015). They are akin to Bayesian predictive tools having varying degrees of correspondence with the external world. They are like islands of meaning (Zerubavel, 1996) that group perceptions of stimuli in the external environment in ways that make sense to the individual and in ways that accord with taken for granted expectations. Using typifications is an interactive process occurring in the immediate present. But it also has to reference past interactions (Schutz, 1970: 218–9). Typifications emerge because of linguistic and communicative exchanges between people and the mutual understandings between them – their intersubjectivity (Schutz, 1967: 10; cf Frith, 2007:139–40).

Ideal types and typifications are used for the pragmatic simplification of complex social life (Di Maggio, 1997:269) and physical and biological environments and are generated neurologically in the same way that all predictive modelling takes place. They are used to solve complex problems in the social, physical and biological environments and to engage in the cultural milieu in which all humans are enmeshed. Typifications help to manage ambiguity and the noise manifest in those webs and are intrinsic to social interaction. The empirical world is highly variegated and complex. There is always more in the empirical universe relevant to the organism than is in the orbit of immediate perception or attention and which will need to be brought into play in order to adjust prior expectations. The brain has to take account of these counterfactuals as well as to make sense of ambiguous and less – ambiguous signals (Seth, 2015). In short, it needs to be both parsimonious and integrative. Typifications are used to achieve this. Typifications help to resist the possibility, and then to minimise the disruptive effects, of external perturbations or signals (Pezzulo et al., 2015:1). The typifications may be thought of as functioning as priors derived from social experience (Schutz, 1970: 118–9) used to make sense of and interpret things – people and objects- around us. They are pre-formed but malleable priors, expectations and predictions derived from ideal types used for developing generative models for predictive processing in the social realm (cf Clark, 2016: 13–29). Inter-individual and supra-individual functioning interact with intra-individual brain processes and, in so doing, models of the external world are developed. The social processes occurring between individuals form a critical substrate for predictive processing.

Typifications and ideal types help to integrate our *a priori* stocks of knowledge about how we anticipate the world will be. They use past experience, vicarious understandings and narrative forms embedded in literature, art, medicine, science, culture, media and religion (Burke, 1937; Zerubavel, 1997: 68–80; cf Clark, 2016:286) as well as the ongoing experience of social practices where typifications are utilised, modified and developed in the webs of interaction of everyday life. Social practices are fluent sets of repeated behaviors flowing across groups of people over time (see below). In the social practices of everyday life, people use ideal types and typifications to make sense of their own and others’ conduct as well as the physical and biological environments in which social practices occur (cf Turner, 2013: 121). Typifications are thus examples of generative models that arise in the social practices of everyday life. Typifications, arising in the networks of social practices link directly to aspects of predictive processing.

Socialisation, which means being recruited into social practices and learning how to execute them competently, is how humans “learn” certain priors about the world, as well as ways of interpreting their own experience. There are shared understandings, which are passed on to the next generation through socialization in education, in families, peer groups, occupational settings and so on. Humans exist in a relational universe in the sense that they exist in networks of relations with other humans and their sense of self, their identity, their social roles and all the things they do – their practices – arise in and are a consequence of relationships. To enact relationships requires, amongst other things, that the person makes judgements about others; about what they are doing, what they might be thinking, and what they are going to do next and in the future, and, counterfactually what they conceivably could have done or might do in the future, differently (Blumer, 1962). Some judgements are made very quickly drawing on heuristics and taken for granted notions (Kahneman, 2011: 21–28). Other judgements are more drawn out and systematically evaluative. Nevertheless, in each case typifications are used to make the judgements and to resolve uncertainty. The human ability to anticipate and interpret the actions of others as a means of facilitating social interaction has been a long-standing interest in social theory (Mead, 1934; Blumer, 1962; Schutz, 1964, 1967, 1970; cf Frith, 2007: 193). The practical consequences of the ability to interpret and anticipate the actions of others is central to the glue that holds social arrangements together (Garfinkel, 1967; Giddens, 1979, 1982, 1984; Goffman, 1963, 1969).

Clark (2013) describing models in the brain, distinguishes between top-level more cognitive models which correspond intuitively to increasingly abstract conceptions of the world (which we have called here ideal types) and which depend on regularities within large temporal spatial scales from perceptual lower-level models which are based on specific kinds of perceptual contact (Clark, 2013: 186) which include typifications. Ideal types and typifications are not only cognitive they are social and where the coalescence between the predictive processing of neuroscience and typifications arising in social practices occurs and the ability to operate in basic motor ways is combined with complex cultural engagement.

It is helpful not to conceive of models in a binary way as either fitting or not fitting the external data. It is more appropriate to think of them as existing on a spectrum of good fit to poor fit with their plasticity allowing reformulation to occur. Humans vary in their capacity to use models, to model experience and to update their models. This is because all models are wrong, but as Box observed, some are more useful than others (Box, 1979). The ability to determine which and how useful the models are, is especially pertinent because this frames when people are able to update models in response to prediction errors and when not to. So individual brains model the external world differently although the processes of modelling are very similar. It is not helpful to think of human actions in binary terms either, as being completely automatic and lacking in any cognitive engagement or as fully reflective and conscious (Di Maggio, 1997: 271). Instead, it is highly likely that actions exist on a spectrum from those, which are largely automatic to those that are largely reflective with many being a mix of automatic and reflective processing. Their placement on the spectrum will depend on the social context (its recursive or changeable quality), the action itself (habitual or novel), intention, and the level at which the predictive processing takes place (whether mostly physiological or mostly social).

## Society and social practices

Typifications and ideal types are intrinsic to social life itself and in the routines and social practices in which people engage everyday of their lives. We suggest that humans are volitional; they are agents of their own actions or at least their sense of self is such that it produces the impression that they are agents of their own destiny (Frith, 2007: 152–5). Obviously, people make many choices and decide to do all sort of things as they go about their daily lives. Some such decisions are trivial; others will be highly momentous. Some will involve detailed cognitive inference others will be hardly noticed because we do them out of habit or apparently automatically (Strack & Deutsch, 2004; D’Andrade, 1995; Di Maggio, 1997: 296). But if we scale up the trillions and trillions of individual choices and actions that are going in the human world all of the time, what we see is that recursive patterns – practices – emerge (Giddens, 1984: 2–19). Certain things happen repeatedly at the level of the social. Certain patterns of human conduct have in other words, a supra-individual relational quality – which sociologists sometimes refer to as social structure. The interaction between individual actions or agency and the social structures produced by agency in turn constrains and delimits in various ways, individual actions and choices in the practices in which they engage.

Emerging out of the interaction between human agency and social structure are social practices – recursive and repetitive patterns of human conduct which exist above and beyond the individual but to which individuals are recruited, participate in for a while and then leave, while other individuals take over the practice, as they in turn are recruited (Shove et al., 2012). Social practices are the interactions within the webs of the material and social structures in which human life occurs (cf Clark, 2016: xvi; Shove et al., 2012) operating at the supra individual level and from which predictive processing flows via the typification process (cf Clark, 2016: 269–76). Some practices are intrinsic to human life like eating, drinking and sexual activity. Some are intrinsic but take on a great many different forms like human work and labour. Still others are historically specific like smoking, enjoying jazz, driving automobiles or using a stone axe, a bow and arrow or a cell phone. Some reflect fashion and changing cultural forms. However, regardless of the practice, in order to engage successfully in a practice, whatever it is, certain materials and object are required and have to be understood and recognised as such. So certain tools, certain competencies and understanding of shared meanings associated with the practices are always necessary (Shove et al., 2012). These are reproduced through the practice itself and in the ways that people talk, think about it, and execute it.

This talk, action and reflection generate the ideal types and typifications, which in turn do two things pertinent to predictive processing. First, they provide a multitude of inputs and signals themselves; second, they provide the raw material for the models – they are the architecture of mind. The typifications arise because of engagement in social practices and are in turn the basis for predictive processing. Clark argues that

“The basic organizing principles highlighted by action-oriented predictive processing make us superbly sensitive to the structure and statistics of the training environment. But our human training environments are now so thoroughly artificial, and our explicit forms of reasoning so deeply infected by various forms of external symbolic scaffolding, that understanding distinctively human cognition demands a multiply hybrid approach. Such an approach would combine the deep computational insights coming from probabilistic generative approaches (among which figure action-oriented predictive processing) with solid neuroscientific conjecture and with a full appreciation of the way our many self-structured environments alter and transform the problem spaces of human reason” (Clark, 2013: 201).

Typifications are the way that the external symbolic scaffolding is made sense of at the social level.

A helpful way to conceptualise the linkage between the neuroscience and the sociology is the concept of the lifeworld. In the phenomenological writings of Schutz (Schutz, 1970: 72–75) the lifeworld is defined as a subjective cognitive space in which the “I” makes sense of the world, renders it meaningful, experiences it through its sense of self in the external environment and shares its subjectivity with others. The things in the here and now and of the moment constitute the innermost zones of relevance of the lifeworld. The innermost zones are surrounded by a series of decreasingly relevant things and people. These may be thought of as a series of concentric rings around the subjective self. Think of these rings of relevance as occupying, metaphorically, a lateral plane. Then conceptualise the generative models described by the neuroscience as occupying a vertical plane crossing in the lifeworld. The higher level models operate at the social level and are derived from the social practices in which the person engages. The lower level models are those which are shaped by the physiological features of the brain’s biology.

While Sociology, with some notable exceptions (Di Maggio, 1997; Franks, 2010; Franks & Turner, 2013; Zerubavel, 1997), has paid relatively scant attention in recent years to the brain, in contrast sociologists have enthusiastically examined the relationship between the body and society (Freund, 1988; Nettleton & Watson, 1998; Shilling, 1993; Turner, 1992). This has produced vibrant empirical and theoretical scholarship which most recently has turned its attention to the embodiment of the social through metabolomic and epigenetic mechanisms (Landecker & Panofsky, 2013). In this paper we are interested in the embodiment of the cognitive through social processes. We have suggested that the neurological processes described by Seth (2015), Pezzulo et al. (2015), Frith (2007) and Clark (2016) amongst others, while quintessentially physiological are embodied in social interaction through the medium of the self. We suggest that the self is in turn social *and* the locus of experience of the body. Our argument flows from the pragmatist tradition which sought to dissolve the boundaries between organism and environment, mind and body, and individual and society (Franks & Turner, 2013:142). Like the pragmatists we hypothesise a transactional and relational process involving separate analytic categories but synthesised in an holistic process.

We are not the first to note the potential commonality between neuro-science and sociology. Others have observed that the writings of Mead (1934), Schutz (1964, 1967, 1970), Blumer (1962), Goffman (1969) and Ralph Turner (1968) speak directly to the core issues of the relationship between the brain and the social world (Franks, 2010: 1–2; Franks & Turner, 2013: 29; Shook, 2013: 37–80). In particular the question of intersubjectivity, or in the contemporary neuroscience idiom the theory of mind (Hopcroft, 2013: 231), the importance of the ability to think about what is in other people’s minds is the very basis of human social and mental life. Mead himself clearly recognised that this was a physiological and neurological process as well as a social one (Franks, 2010: Franks & Turner, 2013: 141–2). The sociological contributions of Mead, Schutz and Blumer have on the whole been overlooked by neuroscientists (Turner, 2013) while at the same time sociologists have tended to keep their distance from the supposed reductionism of the cognitive and neuro sciences (Di Maggio, 1997: 264; Von Scheve, 2011: 255–6). However, several contemporary writers see the opportunity for consilience between sociology and neuro science partly because of the importance of the sociological ideas discussed in this paper but also because, as Di Maggio and Franks and Turner have observed, some cognitive scientists have come to see culture and cognition as supra individual and non-reductionist (Franks, 2010: 2; Von Scheve, 2011: 266–7; Cacioppo & Cacioppo, 2013). Our paper is in that spirit of consilience.

## Implications for power and equity

The idea of predictive processing and of the brain as a model of the world is we suggest highly relevant to issues of inequity and power in social systems. Although the physiology of the brain and predictive processing are universal features of the species, the social environments and the networks of social practices in which people live are certainly not universal; they are highly variegated. Therefore, the higher level modelling processes will not be universal either. Social environments are heterogeneous and social actors engage not only in differing practices, but the practices taking place in different lifeworlds are constrained by class, age, gender, ethnicity, sexual orientation, and geography. These different axes of social difference intersect with each other to create the rich and nuanced web of social life in any given society or culture. The multiplicity of intersections means that the typifications that people habitually use will be very variable according to the complex networks of power, discrimination, disadvantage (or their opposites) which they experience in their lifeworlds. Given the variegated nature of the social world, it seems highly probable that the types of ways in which these social networks constrain predictive processing will also be variable in ways that will reinforce current and habituated patterns of advantage and disadvantage. Ways of thinking constrain ways of living and acting and clearly as decades of research in health, educational, class, gender and ethnic inequalities attest, people’s ability to operate to their own advantage is more optimal for some than for others. The ways that poverty and disadvantage may impinge on executive functioning is suggested by our hypothesis (Marteau & Hall, 2013). Social inequalities are crystallised and then reinforced in the models of the world people use to make sense of it and so to act upon it.

Beyond the impact of class, gender, age and ethnicity and power and discrimination the ways that brain trauma affects cognition and emotion may also be understood by the processes we have outlined. The typifications become distorted, the links between ideal types and typifications are broken with the consequent impact on social action and the ability to participate routinely in social practices gets disrupted in various ways (Damasio, 1994). Likewise addictive behaviors as well as the behavioral and linguistic consequences of intoxication may be illuminated by thinking about the typification processes embedded in social practices. Some forms of psychiatric morbidity (Fletcher & Frith, 2009; Clark, 2016: 73–79) are also explicable in these terms. For some individuals, or for some individuals in some circumstances, there is a reluctance to realign prior models based on experience. This is not just the reluctance that most people have to move out of certain intellectual or habitual comfort zones – the phenomena observed by Tversky and Kahneman (1974) where people prefer their fast thinking solutions even when they do not work very well and lead to errors and mistakes. It is rather the rigid adherence and attachment to models or ways of thinking which produce frank discordance between inner intra-individual processing and inter-individual social processes. If this persists, it may engender a form of model realignment, which is completely lop-sided (Cameron, 1943; Lemert, 1962; May & Kelly, 1992). Also of course, there may be physical or physiological reasons why the signals themselves coming from the internal environment get distorted or the mechanism for generating priors is in some way faulty. In broad terms in both circumstances, psychological distress and social unease and discordance may be the consequence, and the ensuing rigidities and mismatches may generate what come to be seen as mental health problems (Clark, 2016: 71–79).

## Conclusion

George Herbert Mead’s major posthumous work was called *Mind Self and Society* (Mead, 1934) and the title of our paper reflects our debt to him. He set out to show that conscious reasoning by the self was a social process and that the self was itself social – not a physical thing or a psychological trait, state or conditioned response or reflex. Modern neuroscience has hypothesised the intra-individual brain processes by which the brain senses and intends action in a modelled world. By introducing the social environments, we can hypothesise the way that inter-individual processes may work in conjunction with the intra-individual ones (Cacioppo & Cacioppo, 2013). We can go beyond for example accounts of evolutionary psychology to demonstrate the importance of the social in this way; we can describe it in the everyday lives of ordinary people.

The argument is that it is helpful to conceive of human life as a process of continuous interaction among and between individuals engaged in social practices. They are knowledgeable about the practices and their own experiences of them. People are able not just to make sense of the world in a general sense (Giddens, 1984 2–19) but also in ways which feed the very processes of active inference through which predictive processing takes place. In other words, we might think of the processes going on during predictive processing as both individual and as relational/social (Kriznik, Kinmonth, Ling, & Kelly, 2018). Typifications are an emergent property of the interactions between human agency and social structure and the interaction between social practices and the physical and biological environments that are ubiquitous to the human condition (Kelly & Kelly, 2018). The physical sensations of bodily experience as well as affective and subjective feeling states arise in, and are part of, both the interactive processes between people and also the way that they engage in the predictive processing they do. The self is the bridge for these processes as Kant argued more than two and a half centuries ago. It is clearly important to understand the processes going on within the individual as they engage in their predictive programming – as Clark puts it the brain is an inner engine of probabilistic prediction while predictive processing is about how the process is done (Clark, 2016:28). However, it is also helpful to think about the supra individual level and the idea that the very tools which are used in higher level predictive programming emerge out of social interaction in environments which are physical, biological and above all social (Kelly, Kelly, & Russo, 2014).

The external environment and the way it interacts with inter – individual processes is we suggest, as important as the intra- individual processes themselves. The external social and physical world is not just “there” waiting to impact on inter-individual processing and modelling either in a deterministic way or in a process akin to osmosis. Humans actively engage with the external world and in that engagement are constrained by it in various ways. In elucidating those interactions it is possible to describe the mechanisms linking inter and intra individual processes. There are some very exciting accounts of the intra individual processes developed by the authors whose work has influenced this paper like Seth, Frith, Pezzulo, and Clark. In these writings, there are strong echoes of Schutzian phenomenology as well as the interactionism of Mead and Blumer but without reaching out explicitly to these ideas. This paper suggests that these older thinkers’ contributions not only resonate with the idea of the brain as a model of the world, but they help to articulate how the brain through social relationships is mediated by and in turn acts upon the social world.

It is our view that it is important to factor in the role of the brain when thinking about social life. Ideal types and typifications may be thought of as arising in social interaction and as the distillation of the core models used by humans for pragmatic simplification of the complexities of the world as it is encountered. Ideal types and typifications are the means of separating the signal from the noise. They are adaptive because they allow humans to share the models they have in their heads about both what is subjective and what they perceive and understand in the external world. In the course of routine interaction, the models are debated and disputed but they allow for and facilitate human discourse and social action. They are adaptive because they facilitate interaction with other brains. Interaction with other brains is fundamental because ambiguous sense data on its own does not generate symmetry between different brains. The typifications are the priors that facilitate intersubjectivity and probably make social life possible (Shook, 2013:37–8).
